# Medication-free mental health treatment: a focus group study of milieu therapeutic settings

**DOI:** 10.1186/s12888-023-05193-x

**Published:** 2023-10-02

**Authors:** Lise Saestad Beyene, Marit Helene Hem, Elin Bolle Strand

**Affiliations:** 1 Faculty of Health Science, University of Stavanger, Kjell Arholms gate 41, Stavanger, 4021 Norway; 2 Faculty of Health Studies, VID Specialized University, Diakonveien 14-18, Oslo, 0370 Norway; 3 NTNU Social Research, Dragvoll allé 38 B, Trondheim, 7049 Norway; 4 Department of Digital Health Research, Oslo University Hospital, Trondheimsveien 235, Oslo, 0586 Norway

**Keywords:** Focus group interviews, Holistic approach, Medication-free treatment, Milieu therapy, Qualitative study

## Abstract

**Background:**

Medication-free treatment within mental health care aims to offer therapeutic support as an alternative to psychotropic medication. Introducing milieu therapy for severely mentally ill persons in a medication-free unit requires significant changes to the traditional medication-based psychiatric setting. The present study examines how milieu therapists experience working with medication-free treatment for people with severe mental health challenges. The research question was “What may be required to succeed with medication-free treatment in milieu therapeutic settings?”

**Methods:**

A qualitative study with four focus groups were conducted with 23 milieu therapists from three inpatient units in two mental health institutions. Thematic analysis was performed.

**Results:**

One main theme was identified: medication-free treatment involves therapists and patients working together on holistic and personal health promotion. This common thread links the four themes: helping patients to make changes in their life; having time to focus on the individual patient; being a professional companion; and working together as a team with the patient.

**Conclusions:**

A holistic approach is necessary for medication-free treatment to succeed. This requires working together in multidisciplinary teams with a focus on the individual patient. Milieu therapists must engage and take more responsibility in the patient’s process of health promotion. A change from a medical to a humanistic paradigm within mental health care is needed.

## Background

In 2015, the Ministry of Health and Care ordered Norwegian regional health authorities to implement medication-free, user-directed treatment services within mental health care for severely ill patients [1]. The aim of this special assignment was to offer therapeutic support and interventions on a voluntary basis as an alternative to psychotropic medication, including assistance in reducing and ending such medication. The background for the Health Authorities’ initiative was a demand from user organizations to establish medication-free treatment options. It follows a lengthy debate on psychotropic medication’s effectiveness and adverse effects and medication-free treatment [2, 3].

Patients with mild and moderate mental disorders have always had access to non-medication treatments by psychologists or psychiatrists within the public health system or via private practitioners. For patients with severe mental disorders, such as schizophrenia or psychosis, medication has been the most central part of the treatment but is sometimes supplemented with psychotherapy or other psychosocial interventions [4]. A recent review of severely mentally ill patients, who received psychosocial interventions with minimal or no psychotropic medication, revealed huge variation in the content of the interventions [5] and that the effects of the treatments were not significantly greater than for the psychotropic treatments alone [5]. The interventions reviewed included individual or group psychosocial outpatient treatment, with various therapeutic approaches.

A report from 2018 shows that a total of 14 mental health wards with 56 beds have been set up for treatment in Norwegian psychiatric hospitals, most often as part of the ordinary treatment units [6, 7]. The content of the medication-free services is based on the authorities’ professional recommendations and typically consists of individual or group psychotherapy, milieu therapy (MT), art and expression therapy, physical and social activities, and networking related to relatives and work or school. The type of treatment is most often determined in collaboration with the patient and based on needs and wishes for their recovery process.

According to Heskestad et al. [8], about 50% of patients admitted to a mental health ward preferred medication-free treatment if it was available. The authors explain this high rate of preference among patients as due to disappointment in their continued struggle with symptoms, unpleasant adverse effects of medication, or try to avoid the daily reminder of their illness that medication might represent, and the patients’ lack of understanding of their own treatment needs.

Standal et al. [9] revealed that the negative effects of psychotropic medication and unavailable alternatives to such medication in ordinary mental health treatment were the most important reasons for patient’s choice of medication-free treatment. Oedegaard et al. [10] found that important factors for patients undergoing medication-free treatment services included their relationship with the therapist, their own understanding of the pattern of their suffering, and their personal motivation to act in their own recovery process.

Milieu therapy (MT) is recommended as part of the treatment in mental care units in Norway [11]. There are various theoretical perspectives and no uniform definition of MT. The idea with this approach is to create a therapeutic milieu involving “an optimal healing environment based on continuous healing relationships, patient-centred care, safety as a systems priority, and cooperation among clinicians providing a framework to organize care in a holistic manner that supports positive health outcomes” [12, p. 423]. Milieu therapists are perceived as central to patient well-being [13], and research has pointed out that they can play an integral role in successful treatments [14] also because they are involved in daily care for the patients. The everyday interactions between patients and milieu therapists are often seen as trivial and superficial, or considered non-therapeutic as they take place outside formal therapy rooms in everyday-like settings. However, Skatvedt [15] revealed a potential for personal growth even in small day-to-day interactions between milieu therapists and patients. In completely ordinary situations, such as going for a walk or having conversations around ‘everything and nothing’, there can be potential for change. These moments and interactions may contribute to confirming the other as valuable, and building of a patient’s identity as an important or ordinary person [16]. Such seemingly insignificant everyday responsiveness can provide confirmation of belonging, being valuable for the other, and having the potential to ascribe alternative identities [15].

In a qualitative study it was reported that everything in the physical and social milieu affects change [17], and that the environment may function as a therapeutic agent and provide a setting for modelling and practicing behavioural changes. Regardless of theoretical perspective or type of psychosocial intervention, any MT setting has to challenge patients to make changes in their lives, such as how to relate to oneself and cope with challenges in everyday life. Promoting and supporting changes is a difficult task for milieu therapists. Readiness for change is a mental and emotional state within a person and is an important factor for understanding or promoting any changes [18]. There seems to be strong evidence that people with severe mental illnesses can indeed learn skills and make changes in their lives [19]. For example, many patients are just as interested as mental healthy persons in changing physical activity habits [20] even though the prevalence of physical inactivity among mentally ill persons is quite high [21, 22]. Patients vary in their readiness to engage in new activities, such as reducing or quitting medication, becoming more physically active, learning how to handle everyday practical challenges, learning and starting to practice social and interpersonal skills, or changing their level of expended energy or even their identity [23, 24]. Unfortunately, maintenance of acquired strategies are a challenge in all behavioural changes included for patients with severe mental health disorders [18, 25].

Introducing MT for severe mentally ill persons in a medication-free unit requires significant changes to the traditional medication-based psychiatric setting. This is, not at least important for the milieu therapists as they have daily contact with the patients who may need a different follow-up in medication-free treatment than in traditional psychiatric settings where treatment with and the follow-up of psychotropic medication is the main focus. Oedegaard et al. [26] revealed that milieu therapists working within medication-free services experienced Norway’s new medication-free policy as challenging. They found it particularly difficult to balance patients’ needs with treatment guidelines, the legal framework, and available resources.

To our knowledge, not many studies have been conducted into the experiences of MT in medication-free settings. The present study examines in more detail how milieu therapists experience working with medication-free treatment for people with severe and long-term mental health challenges. The research question was “What may be required to succeed with medication-free treatment in MT settings?”

## Methods

### Design

A qualitative study with four focus groups was conducted [27].

### Setting and participants

This study was conducted at one ward at a community mental health centre in Norway, where 12 milieu therapists participated, as well as at two wards at a mental health care institution, also in Norway, where 11 milieu therapists participated. The community mental health centre approached us because they wanted research to be conducted on the new practice they had started couple of years before. Furthermore, we chose to contact the mental health care institution since we wanted to learn from their way of organizing a similar treatment option. By such a procedure, we secured a larger selection of participants and the possibility of creating a richer data material. Both sites were within easy reach of where we operate.

The community mental health centre is responsible for a geographical region of the country and offers specialized treatment for various forms of mental health issues and substance abuse problems. Patients may use different forms of psychotropic medication. The treatment approach is inspired by cognitive therapy where the aim is to help the patients find new ways of approaching their mental health and/or their substance abuse problems. The centre consists of several departments, of which the participating department has an assessment and treatment service with 10 beds. The treatment is based on voluntary admission. Patients can be referred by a GP, a specialist, or other treatment units in the region.

The mental health care institution offers recovery-oriented treatment both for people who do not use psychotropic medication and for people who to varying degrees use different types of psychotropic medication. When patients want to reduce or stop the medication, arrangements for this are made. The aim of the treatment is to help people to live good and independent lives without unnecessary or excessive use of medication. The mental health care institution consists of two departments with 60 beds.

The milieu therapists in both the community mental health centre and the mental health care institution are responsible for the therapeutic milieu within the ward. In the community mental health centre, the milieu therapists worked in a three-part rotation. In the mental health care institution, they organize MT based on so-called ‘co-living rotation’. This means that the milieu therapists are organized into teams that are on duty for several days in a row. They live with the patients for 14 h at a time – they eat together and exercise together – and often engage in long conversations with the patients. After seven days, they hand over responsibility to the next team for another seven to 12 days. The continuity of this co-living rotation is meant to contribute to good communication and the establishment of trust between the milieu therapists and the patients.

Participants were recruited by the head of the departments. The inclusion criteria to participate in this study were milieu therapists who had experience working with patients in the context of medication-free services. The selected therapists (n = 23) were aged from 24 to 60 years. They consisted of six males and 17 females who had from a couple of months to 25 years of experience in mental care inpatient settings. They included mental health nurses, registered mental health nurses, social workers, pedagogues, and auxiliary nurses. All the participants included in the study were unknown to the authors.

### Data collection

The data collection was conducted by means of four separate focus group interviews. The focus groups were put together by colleagues at the same institution without everyone necessarily knowing each other well. Focus group interviews – through the group reflections and discussions—tend to yield rich data concerning the experiences and perspectives of various stakeholders [28]. Two of the interviews were conducted in June 2020 and the other two in June 2021 by the second (MHH) and the last (EBS) authors. The focus group interviews were conducted at the community mental health centre and the mental health care institution where the participants were employed, in a room separated from the wards where they worked. Each session lasted for about 60 min. The main focus of the discussion was the participants’ experiences of practicing medication-free services in indoor mental care, reflecting on the settings from their everyday practice. All interviews were audio-recorded, held confidentially, and kept securely locked away [29].

### Analysis

A thematic analysis of the qualitative data was conducted by all authors. Inspired by Braun and Clarke [30] we identified themes, i.e. patterns in the data that were of interest to illuminate our research question regarding what may be required to succeed with medication-free treatment in MT settings. A thematic analysis, moving back and forth between the various stages, made it possible to answer our research question in a thorough way (ibid.). At stage one, we familiarized ourselves with the data by reading the transcribed interviews several times. In the second stage, initial codes were generated and organized into groups. An example of a code is: *being available.* At stage three, empirical patterns were identified. Similarities and differences between the codes within the data set were searched for and compared, which gave direction for the codes to be sorted into pertinent groups labelled according to preliminary themes. During the fourth stage, a validation of this abstraction was performed by reading the text as a whole to explore if the themes articulated the pattern and if they reflected the codes and data. At stage five, patterns of the themes were refined and named, e.g. ‘*being a professional companion’*. Each separate theme is a necessary part of the whole but is not sufficient on its own to contribute to a deeper understanding of what may be required to succeed with medication-free treatment in MT settings. In the sixth and final stage, the authors went beyond the original content to interpret the pattern of the themes and identify the overall theme: Medication-free treatment is about working together on holistic and personal health promotion [30].

### Ethical considerations

The work was undertaken conforming to the provisions of the Declaration of Helsinki [29], which means that basic ethical principles for research ethics such as informed consent, the right to privacy, and respect for personal integrity and dignity [31, 32] were followed. All participants gave informed consent after having received written and oral information about the project. Participant and patient anonymity are preserved in the text. The protocol for the research project has been approved by the Norwegian Social Science Data Service [33], where aspects of privacy protection were assessed (approved 14 November 2019; NMB: 141,235). Since the study does not include patients as participants, we were not, according to Norwegian regulations, obliged to seek approval from the Regional Committee for Medical and Health Research Ethics.

## Results

**Fig. 1:**
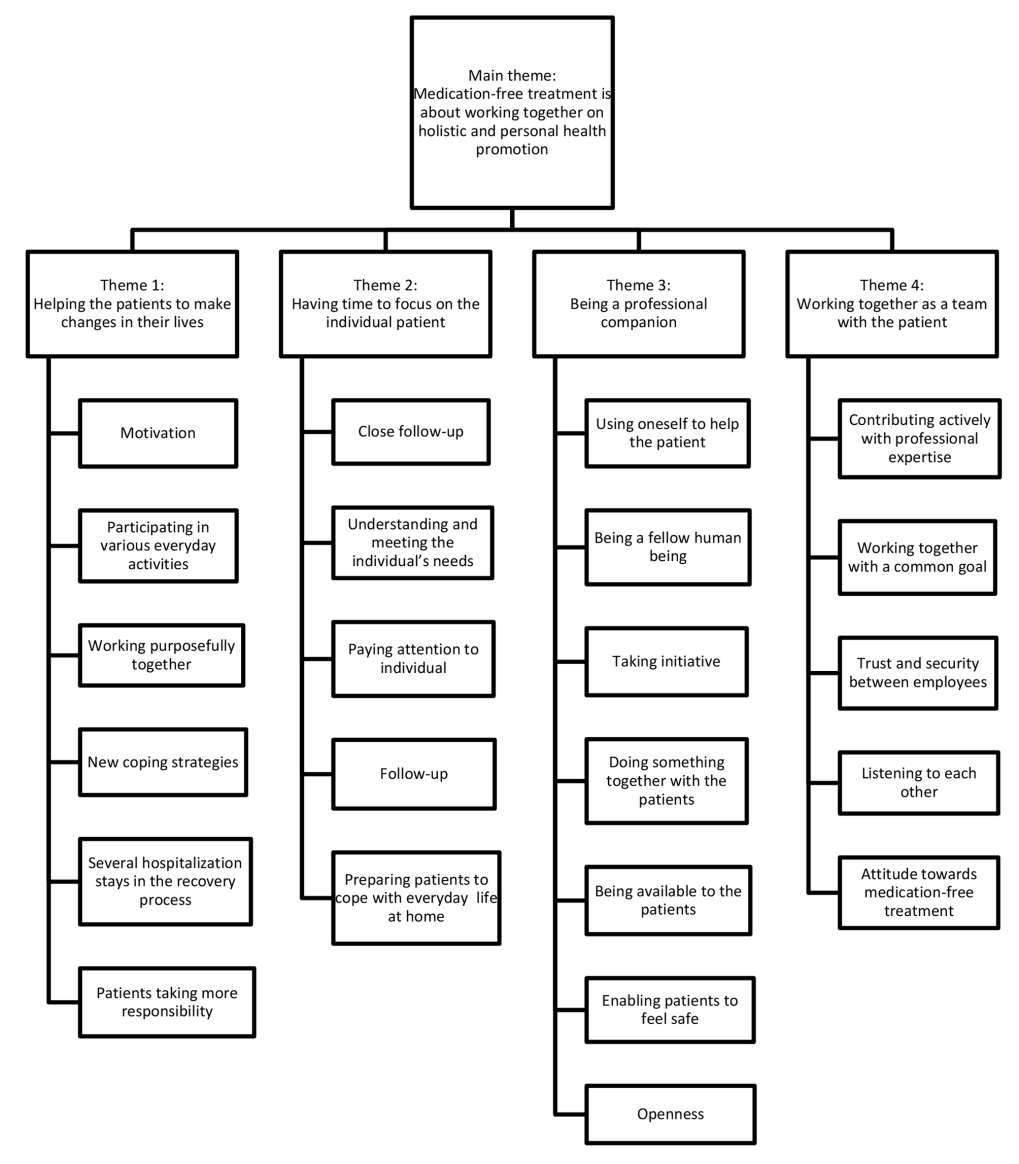
Code tree with main theme, themes, and codes

One main theme was identified: Medication-free treatment is about working together on holistic and personal health promotion, which represents the common thread linking the four themes: helping the patients to make changes in their life; having time to focus on the individual patient; being a professional companion; and working together as a team with the patient (Fig. 1). Throughout the analysis it was revealed that the participants from the community mental health centre conveyed that they did not experience success with medication-free treatment, i.e., that they did not experience that their patients were well enough looked after with the service they offered their patients, and they did not experience to succeed in helping the patient to reduce the use of psychotropic medication. The participants from the mental health care institution were dedicated to medication-free treatment, and they communicated that they experienced success with this treatment, i.e., they experienced that the patients developed a higher level of functioning when they were hospitalized, using less or no psychotropic medication. Answering the research question *what may be required for medication-free treatment to succeed in MT settings* we present the pattern within the themes through the participants’ experiences of succeeding or not succeeding with medication-free treatment.

### Medication-free treatment is about working together on holistic and personal health promotion

The participants in this study, who all had experience of offering patients a medication-free treatment option, described how receiving this treatment might help patients cope with life with their mental illness:

When you come here, the focus isn’t “how can you become medication-free?” The focus is on what does it take for you to have the best possible life?

Psychotropic medication could be part of but did not play a central role in the treatment. The experience of the participants was that psychotropic medication can often complicate contact with patients, and they often saw that when patients reduced their use of such medication, they became more open to contact with themselves and others and that it became easier for patients to work with changes. This is important in order for the patient to be able to benefit from a treatment option that focuses on getting to know themselves in a new way, becoming aware of what they need, seeing their life as a whole in their recovery, and learning new strategies for coping with everyday life:

*It’s about getting to know themselves on another level, so they can cope better with everyday life when discharged*.

At the same time, participants found that some patients need medication to become mentally available for other treatments.

### Helping the patients to make changes in their life

The participants found that patients have varying degrees of motivation to make changes in their own lives when they are hospitalized. It became important to help them see their own situation and what they need.

Many patients lacked a fixed structure in their everyday lives because of their mental illness. Participants expressed the opinion that it is essential for patients to participate in various everyday activities during hospitalization in order to work with holistic recovery processes. Various physical activities were highlighted as central to this work, while diet and nutrition, the course’s Illness Management and Recovery programme, cognitive therapy, music therapy, and art therapy were also deemed important.

The participants who did not feel successful with medication-free treatment said that they often took a dominant role in helping patients make changes in their lives. They described that they struggled to persuade very ill patients to accept medication or increase the dose:

*We may be struggling to get in the position that they should accept medication or budge on the dose they may have because they are so ill*.

These participants also found it challenging to work with the patients toward a common goal:

*It may not feel like you have a joint project with the patient*.

On the other hand, the participants who experienced success with medication-free treatment said they worked purposefully together with the patient, based on the individual patient’s wishes and needs:

In conversation with the patient, we write goals and measures; what we are going to do through the programme, and what is important for him or her. During the four months [during which the patient is admitted] we repeatedly review goals and measures and evaluate and write. Is it progression or not, and what should we work more on? We do all this in collaboration with the patient.

The participants found that the changes achieved by the patients or new coping strategies they learned during hospitalization could be difficult to maintain after admission. Those participants who experienced success with medication-free treatment were aware that they were working in the process together with the patient. They worked to increase the patient’s awareness of what might happen, and together with the patient made concrete plans for how they could prevent relapse and maintain changes they had initiated, when they returned home to everyday life.

Making changes in life requires a person to experience new ways of coping with everyday life. The participants who reported they succeeded with medication-free treatment worked actively to ensure that patients had their own experiences. They found that while many patients were well on their way to recovery at discharge, some needed more hospitalization stays to continue working. They found that medication-free treatment must be worked on over time. This might require follow-up with patients over many years as well as possibly future hospitalization stays, which should be seen in this context and followed as a process.

Those participants who had experienced success with a medication-free treatment programme found that an important prerequisite for improvement was that patients took action themselves and were able to take responsibility for their own choices and life. They saw it as important to make sure that the patient had space to figure things out on their own. They also arranged for the patient to ask for help when he or she needed it, to make an effort to work on their own recovery process, and to contribute to helping others or providing understanding to fellow patients. The participants found that not all patients wanted or had the energy to make this effort themselves and that some patients found themselves to be unfamiliar, frightened, or lacking the confidence to take responsibility themselves. They explained that they then spent a large amount of time helping to ensure the patients had faith in being able to take on more responsibility, thus motivating them to make changes in their lives.

### Having time to focus on the individual patient

When the focus on, and or the use of, psychotropic medication is removed, it is essential to be able to offer something else instead. The participants found that offering a holistic aspect to the treatment, without relying on the effect of psychotropic medication, requires that they spend a large amount of time and focus on the individual patient. The participants whose working hours were structured through co-lived rotation, working several days in a row while living together with their patients, experienced close follow-up, spending everyday activities together with their patients. They got to know the patients well and saw what they needed to recover.

The participants who did not experience success with a medication-free programme described few MT approaches beyond physical activity and giving patients a safe place to rest, as their main focus was on medication tapering:

*The goal is somehow to get rid of the medications, versus finding a way to live without medication (…) When people are admitted to medication-free treatment, the focus will be, for example, the reduction of medications. Also, that’s fine, but I don’t want to. It’s mostly not the medication the patient has a problem with. In other words, there is an underlying problem that the patient is taking medication for*.

Participants found that if they were to succeed with medication-free treatment, they had to see, understand, and meet the patient at an individual level. This meant that they became well acquainted with the patient personally and had the opportunity to adapt based on individual needs and differences. To get to know the patients well, the participants found that they had to be available by being physically present, making it easy for patients to contact them when needed. They found that it was important that they, as milieu therapists, took the initiative to talk to the patient, both formally and purposefully, but also informally. Being where the patients were physically meant that they could pay attention to and follow up in situations that were challenging for the patients. This also required them to be emotionally present and able to capture and understand how the individual patient was feeling. They found that spending their time directly with patients was necessary to succeed with medication-free treatment. By contrast, the participants who did not experience success with medication-free treatment reported that they spent a large amount of time on administration, and they spent little time directly with the patient:

*We do not have one-to-one contact*.

Professionals who experienced success with medication-free treatment worked with a focus on preparing patients to cope with everyday life at home. This meant that they engaged in the patients’ lives, their families, and networks, that they had a holistic focus, and worked closely with the patients:

*We can’t just talk to the patient. The family must be involved, and they must be given time to get involved in the patient’s recovery. Then we can attain good cooperation*.

Each patient had ownership of the treatment course and treatment plan with their own goals and action plans which were evaluated and adjusted regularly. Those participants who did not experience success with medication-free treatment referred to working mainly based on the doctor’s prescription. They did not have the opportunity and time to familiarize themselves with the individual patient’s needs, but they tried their best to find a good treatment:

*We haven’t spent a lot of time familiarizing ourselves with reality or been given the opportunity to do so .*. *we try our best*.

### Being a professional companion

For those who experienced success with medication-free treatment, one of the most important aspects of the approach was to be a professional companion. This meant that the milieu therapists stood on an equal footing with the patients and saw themselves as fellow human beings who used themselves, their knowledge, and their experiences in how they supported the patients. They actively participated in patients’ everyday lives and followed them in their recovery process:

*Milieu therapy becomes much easier when we work where we live. Then it becomes more natural because we blend in more with their everyday life here. We eat and we exercise, and we talk with the patients.*

Although the patients themselves took a large share of responsibility and had good control over what was right for them in the treatment, the participants were active as therapists by taking the initiative, motivating the patients, asking questions, and giving them a friendly push in the right direction:

*Some need a little more motivation and kind of need to be pushed a little bit*.

Doing something together with the patients, such as physical activity, group activities, or practical tasks, was highlighted as important.

Participants who experienced success with medication-free treatment conveyed that they were conscious of being available to the patients, and there was a low threshold for patients to be able to contact them. They found it necessary to have a good relationship with the patients to be in a position to be able to help them in their recovery process. They pointed out that it was necessary that the patients felt safe with them, that there was openness between them, and that the patients felt that they were seen and cared for by receiving the necessary information and knowledge. It was also important to provide good help and support, balanced with the patient’s independence and responsibility.

### Working together as a team with the patient

MT wards are interdisciplinary, and the participants found that it was essential that the various professional groups contributed actively with their expertise and could work together to think holistically in the practice of a medication-free treatment programme. Those participants who experienced success with medication-free treatment said that the various professional groups together formed a team that worked together with the patient, towards a common goal related to the patient’s overall recovery process. They worked closely together, knew each other well, were available to each other, and contributed to good communication and the establishment of trust and security between the employees:

It allows us to understand how each one of us works, and in what ways we work. What each of us is good at, who is stronger in what, and who needs more guidance, which makes us very good at allocating tasks based on what we are good at, strengths we have, and what we think is okay or not.

Professionals who did not experience success with medication-free treatment often found that they did not know their colleagues, that coordination of the services was often lacking, and that different therapists could have different approaches:

The music therapist has a project, the sports educator has a project, the psychologist has a project, and then we (the milieu therapists) have a project in a way, and then the patient maybe has a completely different project. And then you compete for time to run your project.

The participants also felt that they had too little knowledge about the medication-free services they were supposed to offer patients, while at the same time, the patients received insufficient information about the medication-free treatment. They conveyed that it was problematic to implement such a treatment option in a medical system where the therapist and the head of the treatment were doctors with medical expertise, and where other professional groups were intended to follow the doctors’ decrees. Those participants who did not experience success with medication-free treatment felt a lack of understanding and a lack of trust between them and the doctors and found that the doctors did not listen to their knowledge, observations, and point of view. This made it difficult to work together as a team with the patient.

The participants said that it was important that they themselves had a good understanding of the medication-free treatment they were to be a part of, in addition to having a fundamentally positive attitude towards it.

## Discussion

The present study examines how milieu therapists experience working with medication-free treatment for people with serious and long-term mental health challenges. We asked what may be required to succeed with medication-free treatment in MT settings. The main finding was that medication-free treatment requires working together on holistic and personal health promotion. This consists of helping patients to make changes in their life, having time to focus on the individual patient, being a professional companion, and working together as a team with the patient.

### Helping the patients to make changes in their life

In order to succeed with a medication-free treatment, participants highlighted that an important part of the treatment is to assist the patients in learning new strategies for coping with everyday life and, through conversation, to motivate them to implement changes. It is one thing to be motivated to reduce or stop taking psychotropic medication, but it is quite another to start new activities in your everyday life, for example changing your diet, or becoming more socially or physically active.

The purpose of the intervention is to motivate, facilitate, and push the patient further in a process of taking more responsibility in their own lives. Patients with severe mental illness are less active than others [17, 34], and most of the patient group are thought not to be ready to make such changes [35]. It is therefore understandable that motivational work is central to the success of medication-free treatment. Even if therapists are unable to persuade patients to make changes during and after their stay, hopefully those individuals have after all gained some new and positive experiences about themselves. The close and continuously contact between the milieu therapists and the patients, as well as the milieu therapists’ availability during the stay, may provide ample opportunities for everyday responsiveness for the therapists and thus personal growth for the patients [16]. In a sense, confirming the patient’s value can help to build the patient’s identity as a significant human being [15, 16].

Motivating others to change is generally challenging and may require specific knowledge and practical skills. This study only reveals to a limited extent how milieu therapists actually work, except that they are available for conversations much of the time. There was little focus on which specific therapeutic approaches and methods they utilized. Close follow-up, such as the availability of milieu therapists during the stay itself, has proven to be an important factor for the success of medication-free treatment [10]. Availability may also be of importance after a patient’s stay because when they return home and are faced with the challenges of maintaining their new skills in everyday life, the risk of relapses to old habits is considerable. Strengthening continuity of care, it would be preferable to give patients the opportunity to contact the institution after discharge for support and follow-up as well as to apply for repeated stays if necessary [36, 37].

### Having time to focus on the individual patient

The present study showed that an essential element to the success of medication-free treatment in a MT setting is having time to focus on the individual patient. This is in line with person centred care, always setting the individual patient and his or her views and needs at the centre of care [38]. Having time to spend with each patient is an important base for building therapeutic relationships [10, 39]. Oedegaard [26] report that therapeutic relationships between therapists and patients involving information-sharing, trust, and availability are essential for succeeding with medication-free services.

Having time to focus on the individual patient is important in medical-free services. However, how the time with the patients is spent, as well as the attitude of listening to the individual patient, are more important than time in itself [39]. The content of the time spent with the patients should be constructed on a tailored basis [40]. In alignment with person-centred care principles, the patient is recognized as an integral participant in their own care. It is essential to consider the unique personal attributes, capabilities, strengths, aspirations, and interests of each individual when providing treatment [38]. It is therefore important to have enough time to get to know each patient and to understand their life and views to help them in the process of restoring their mental health. Focusing on the individual patient requires acknowledging them as a person, which is essential for mental growth [41]. This is in line with Oedegaard et al. [10], who point out the importance of including the patient’s own understanding, their personal coping strategies and more personal responsibility in medication-free treatment. Existing practice is often characterized by efficiency and a focus on following the guidelines, which is a challenge to having time to focus on the individual patient and introducing medication-free treatment [10]. As presented in the results, having time to focus on the individual patient involves the milieu therapists to be emotionally present and able to capture and understand how the individual patient feels. This requires them to be conscious and determined in everything they do together with the patient. At the management level, this means that there must be sufficient professionals at work, and that work routines and the daily rhythm are adapted primarily to the patients’ need for focused time together with their milieu therapists.

### Being a professional companion

When suffering from mental illness, most patients are emotionally distressed with high emotional pressure. They may be anxious, fearful, depressed, and angry, and they may have difficulty controlling their emotions. Often these emotions will be transferred to the milieu therapists. Some patients may lack insight into their own situation, and some are not able to take responsibility for their own actions. Psychotropic medication is used to stabilize the patients, and to help them deal with their emotional pressure. In medication-free services, it is vital to replace psychotropic medication with therapeutic relationships [10].

This study’s results highlight the importance of milieu therapists taking the role of being a professional companion. By doing so, the therapists can support and encourage their patients when working to improve their mental health by cooperating and contributing with their own professional expertise for each individual patient’s benefit. This implies that milieu therapists should be involved in a dynamic process together with their patients and at all times understand their needs [41]. They must not only respond to the pathology but also exercise problem-solving, empathy, hope, and self-awareness in order to safeguard the best possible conditions for their patients’ recovery process [42].

This in turn requires close attention to emotional and relational skills. Milieu therapists must be able to reflect, react to, and understand different situations in a professional manner [43]. It is also important that milieu therapists are able to process emotional information in a professional way. This involves the mental capacity to be aware of one’s own attitudes towards emotions, to be able to distinguish between certain emotions, and to have good emotion-regulating strategies [42]. High emotional and relational skills are therefore required of milieu therapists in order to be a professional companion and so to succeed with medication-free treatment in an MT setting.

### Working together as a team with the patient

One aspect of working together as a team was that many milieu therapists felt that they contributed with their expertise. This meant that the therapists recognized the differentiated competences of their colleagues working with patients in a medication-free treatment alternative. Mutual recognition and respect between colleagues created trust in the MT settings. Mutual respect between colleagues also entailed having insight into each other’s competence. Trusting relationships in which employees cooperated well, among other things listening to each other, enabled mutual understanding of colleagues’ competence. This was essential to build ‘a consistent and structured environment’ [17] – a therapeutic milieu – where the patients could explore themselves, such as trying out new ways of handling themselves and the challenges they faced within the framework of a medication-free treatment option [17, 44].

A therapeutic environment involves a social climate where the relationships between patients and milieu therapists are supportive [45]. A study by Andersson [46] shows that key characteristics of supportive relationships are that health personnel show interest in the patient’s individuality, that they demonstrate concern and care for the patient, and that they respect the integrity of the patient. In other words, the social climate is of importance for treatment outcome. When the social climate in the ward is characterized by mutual trust and respect, this means that patients cooperate and help each other in the personal work each one must do to recover.

The teams that were characterized by a lack of cooperation were unable to settle on a common approach toward the patient. There was also a lack of managerial involvement and anchoring of medication-free treatment services among the various professions working at the wards. In such cases they typically lacked ‘an ethos’, i.e. a common perspective or ‘culturally based way of viewing the world – a collective set of beliefs and values that guide practice’ [17, p. 112]. In such an environment, patients are unlikely to be able to help and support each other in the process towards recovery.

Another aspect of working together as a team with the patient has to do with the ways the milieu therapy was organized in the institutions. In the mental health care institution where the milieu therapists worked in teams being on duty for several days in a row – the so-called co-living rotation – they could slow down, getting to know the patients and each other as colleagues. This created continuity which supported the patients’ process towards recovery. This way of organizing the milieu therapy was anchored in the entire institution. (However, they faced challenges regarding the continuity between the different teams). In the community mental health centre where they organized the milieu therapy as a three-part rotation, and where medication-free treatment was not institutionalized in the same way as in the other institution, this seemed to inhibit what milieu therapists managed to do and achieve. How organizational differences facilitates or inhibits the practicing of milieu therapy is an important area for future research.

### Strengths and limitations of the study

One strength of our study is that we cover experiences from those who, according to their own viewpoints, had succeeded with medication-free treatment, as well as those milieu therapists who struggled with and were critical about establishing such a milieu treatment. As a result, this study adds knowledge concerning which conditions should be in place to successfully offer medication-free treatment. However, although the milieu therapists tell us what they can do to succeed, we know nothing about the extent to which they actually do it, or the effect of the interventions in the long term; for example, whether patients change their behaviour, take more responsibility, or become more active in certain areas when coming home. The fact that we included only milieu therapists and neither psychologists, psychiatrists, nor patients is both a limitation and strength: we wanted to learn about how milieu therapists perceive medication-free treatment, which we did, but we did not learn about the viewpoints of the other stakeholders. Participant observation, in addition to focus group interviews, could have yielded even more differentiated knowledge about medication-free treatment, for instance how patients perceive relationships with the milieu therapists. Our approach yielded rich data on how milieu therapists struggle with challenges in everyday clinical practice. However, by choosing another research question and a different approach to the analysis, we could have highlighted different aspects of this rich data material like the organizational/structural aspects of implementing medication-free treatment. Furthermore, it is possible that a more explicit theoretical framing of the project from the outset could have led to a clearer conceptualisation of ‘medication-free treatment’. We did not learn about which specific therapeutic perspective, methods and strategies were used by the milieu therapists. This could have been explored or requested more in the interviews. Finally, there is a potential limitation related to the recruitment done by the head of the departments, which may have led to a narrow sample.

## Conclusion

The present study examines how milieu therapists experience working with medication-free treatment for people with serious and long-term mental health challenges. We asked what may be required to succeed with such treatment in MT settings. Our results show that a holistic approach is necessary to establish a medication-free treatment. To succeed with medication-free treatment in MT settings, milieu therapists must help the patients to make changes in their life, have time to focus on the individual patient, be professional companions, and work together in multidisciplinary teams with a focus on personal health promotion for the individual patient. Milieu therapists must engage with and take more responsibility in the patient’s process of health promotion. This may challenge the dominating medical paradigm within mental health care.

## Data Availability

The dataset analysed during the current study are not publicly available due to the anonymity of the participants but are available from the corresponding author on reasonable request.
